# The Deficits of Students’ Orientation in Solving Proportion Problems, as Revealed through Task Modifications

**DOI:** 10.11621/pir.2023.0303

**Published:** 2023-09-15

**Authors:** Maria A. Yanishevskaya

**Affiliations:** a Psychological Institute of Russian Academy of Education, Moscow, Russia

**Keywords:** Concept acquisition, ratio concepts, assessment, task modification

## Abstract

**Background:**

Using the Activity Theory of education (Galperin, 1992; Talyzina, 2018), this article examines the students’ actions that constitute the early stages of forming the concept of ratios. The psychological analysis of mastery of this concept shows that it essentially depends on understanding the coordination of the changes of two independent values (area, velocity, density, etc.).

**Objective:**

The present research considers differences in students’ operations with numbers on various tasks, based on their comprehension of ratio relations (direct and inverse proportions); these differences are revealed through posing certain modified tasks, but may stay unnoticed in regular tasks. The goal of the study was to identify the criteria for adequate assessment of the sustainability of the students’ orientation in modified tasks.

**Design:**

A test of 15 tasks was designed based on Galperin’s classification of task variations: domain specific, logical, and psychological. The formulation of the tasks disguised the operations needed to achieve the right answer, and sometimes even prompted the wrong solution. There were 12 tasks on direct proportions — four sample and eight modified; and three inverse proportion tasks: one sample and two modified. One hundred sixty (160) students (5–6th grade, 11–13 years old) took the test in writing.

**Results:**

The comparison of students’ performance on the sample and modified tasks showed significant differences. Modifications impaired the students’ performance on both types of proportion problems (direct and inverse). Logical and psychological modifications had the most impact on the quality of the students’ orientation and thus proved to be most indicative in terms of students’ orientation quality assessment.

**Conclusion:**

The data suggest the following: 1) that the concepts of proportionality which the students acquired from a regular school curriculum lack “generalization,” and 2) that students’ ability to apply the ratio concept is very sensitive to the way the word problem is presented. These findings are essential for evaluating students’ multiplicative thinking: their actual level of comprehension cannot be revealed through their performance on regular tasks.

## Introduction

The Activity approach in education (Davydov, 2008; Galperin, 1992; Talyzina, 2018), as presented in most prominent theoretical and experimental works, demands to consider the basic concepts and their orientation meaning for designing one’s own solution (instead of only acquiring “universal” skills and specific individual techniques). Being able to evaluate the quality of concept formation as the result of learning is hence an important goal within this approach. In the current research we explored some typical mistakes which emerge in calculations related to the concepts which imply consideration of two values comprising a ratio.

Since Piaget, ratio-based concepts have been at the center of psychological research (Inhelder & Piaget, 1958; Siegler, 2013; etc.). Students’ application of such concepts (buoyancy, concentration, area, work, density, velocity, pressure, exchange rates, etc.) tends to be “troublesome” throughout school disciplines (Hecht et al., 2007; Lutfi et al., 2023; Obukhova, 1968). Mastering these concepts is vital for many school subjects, and at the same time these concepts are considered indicators of one’s psychological development (Rubtsov, 2021).

Difficulties in using ratio-based concepts may impact students’ advance in different discipline areas. Both psychologists and educators are in search of ways to prevent, or at least overcome, students’ difficulties with learning ratio concepts (*e.g.*, Lamon, 2012; Lobato & Ellis, 2010; Matthews & Ellis, 2018; Watson et al., 2013). For example, Simon and Placa (2012) point to the inadequacy of introducing such concepts using “one-dimensional space;” they discuss the benefits of a “two-dimensional space” in presenting ratio problems. Still, the in-depth study of the mechanisms behind mastering ratio concepts remains a challenge.

Since we use the Activity theory (Chaiklin, 2019; Engeness, 2021; Galperin, 1992; Galperin & Talyzina, 1957, Podolskiy, 2022) and Developmental Instruction approach (Coles, 2021; Davydov, 2008) to teaching and learning at school, our interest focuses on the quality of concept formation during the transition period from primary to secondary education, when the children need to master mathematical concepts for the standard curriculum (11–13 years). Our previous studies on ratio concepts (Vysotskaya et al., 2017; Vysotskaya et al., 2020; Lobanova et al., 2022) outlined a feasible way to scaffold their formation: a number of teaching strategies (contexts, learning situations, models, etc.) and approaches to educational design were devised.

Since we planned to test all these ideas together within a teaching experiment, our first challenge was to devise the appropriate diagnostic tools. The widely used diagnostic procedures for assessing proportional reasoning include clinical interviews concerning problem-solving (Empson, Junk, Dominguez, & Turner, 2006; Inhelder & Piaget, 1958), individual written tests (Dole et al., 2012; Hilton et al., 2013; Martínez-Juste et al., 2023), practical tasks, and exploiting operations on magnitudes, distributed among participants (Rubtsov, 2021). However, the challenge of finding and designing indicative tasks remains urgent.

Talyzina (2018) highlights intellectual development itself as the core content to be diagnosed. Karpov & Talyzina (1989) outlined two basic principles for evaluating the level of students’ mastery in some domain. First, it is necessary to identify the cognitive actions which lie behind the concepts. Second, one needs to select tasks that allow you to assess the main parameters of these actions: form, generalization, consciousness, mastery, etc. (Karpov & Talyzina, 1989). This approach to diagnostics, deeply grounded in Activity Theory, has great potential to improve the quality of the educational process, by focusing on concept formation.

The present study focuses on one of these parameters — generalization — which indicates the sustainability of the “concept-mediated” orientation formed. Galperin described “generalization” as follows: “Therefore, the idea of generalized action means that the acting subject (the learner) is able to identify the significant conditions for the particular action among the variety of conditions in which he operates. … Therefore, a learner has to demonstrate stability, a degree of insensitivity to any interference, and be able to identify the significant conditions needed to perform the action” (cited by Engeness, 2020a, p.6).

Galperin (Engeness, 2020b) and Talyzina and Karpov (1989) suggest a feasible way to test “generalization” using task variations. An action is “generalized” if it can be performed under conditions that vary and sometimes are quite disruptive. According to Galperin (Engeness, 2020b), the tasks should vary in three ways: 1) the particular actions that help to solve the problems should be different; 2) the problems should include excessive information or be deficient; and 3) the problems should challenge seemingly obvious and standard conceptual features. The modifications of task conditions may thus involve:

Domain-specific types of material. This kind of modification focuses on the conceptual part of the task: what should be done to solve the problem? Merely varying numbers in a math problem does not constitute creating different types of tasks.

Logical variation means that there can be more data than is needed to solve the problem or/and some important data can be missing. Thus, there are four types of problems. First, most problems will contain data sufficient to solve the task, and there need be no excessive information. Second, there can be more data than is needed so that a student has to make some extra effort not to be distracted by irrelevant data. Third, some vital information may be missing, so that the task can only be solved in general terms. The fourth type of variation involves both extra and unnecessary data and the lack of necessary information.

Psychological variation relates to the process of solving the problem. Vividly represented and conceptual features of the material may either coincide or diverge: the appearance of the task (the wording, the order of data presentation, illustrations, etc.) itself directs students and give them hints as to what to do to get an answer — either correctly or incorrectly (for example, what arithmetic operations are implied). N.F. Talyzina and P.Ya. Galperin (1957) specially emphasized the importance of this task modification for evaluating the students’ level of generalization while teaching — and for distinguishing between different levels of generalization in diagnostics.

We designed the tasks with variations to “maintain the tension” during concept acquisition: each task differed from the previous one, so that the students always had to reconsider the basis for their actions. At the same time such variations could also be used to evaluate the state of the students’ concept development and the quality of their actions. The students’ mistakes were considered signs of some deficiency in the orientation basis of their actions.

Many students who learn to solve ratio-based problems tend to produce a number of “roundabout” ways to succeed without applying “conceptual” understanding. Thus, an assessment should distinguish between the appropriate orientation concept-mediated procedure and “formally” correct solutions based on some random irrelevant features, which are enough for solving trivial sample tasks. Galperin’s and Talyzina’s approach to meaningful variations of task conditions should thus be productive for diagnostics design in this domain. The goal of our study was to identify the criteria for adequately assessing the sustainability of students’ orientation in solving ratio-based problems through tasks modified in this way.

## Method

The research question was to assess the current quality of mastery of the proportionality concept by 5–6th grade students, since it is crucial for problem-solving in Math and for future promotion in the Natural Sciences.

One hundred sixty (160) students (5th-6th graders, 11–13-year-olds) of high and medium academic level from three urban schools participated in the study. The pen-and-paper test consisted of 15 tasks: 12 tasks based on direct proportion (“the more, the more” — type D) and three tasks based on inverse proportion (“the more, the less” — type I). The assessment was conducted in individual written form during a regular Math lesson. It took from 30 to 50 minutes to complete the assignment.

The tasks on the test were designed according to the typology of modifications introduced by Galperin (see Introduction):

1. Domain-specific task variations required diverse sequences of arithmetic operations to solve the problem, included numbers that were “inconvenient” for calculations, and presented data in different formats — pictures, dialogues, and diagrams.
2. The tasks with “logical” variations included only the necessary data or necessary data with excessive information. We did not suggest tasks with missing data, as they demand more in-depth analysis of students’ solutions.
3. The tasks were “psychologically” varied through the contrast between their “vivid” and “conceptual” conditions. Thus, some of the modified tasks misled students to wrong solutions based on “eye-catching” details.

The majority of tasks contained one or more of the modifications listed above (type M, modified). Four tasks for direct proportions and one for inverse proportions contained no modifications (type S, “sample”). They were used as the reference points; students’ performance on the other tasks were compared to the sample-tasks. The tasks below exemplify the modifications that we introduced.

The “shell” task was the “sample task”: it exploits a familiar context; obvious operations lead to the right answer, and the calculations are simple.

“Shell” task (№ 1), direct, sample (DS): Serge and Mike exchange pebbles and shells. They agreed that 4 stones can be exchanged for 5 seashells. How many shells will Mike get if he offers 12 stones?

The “coal” task contains the simplest math-specific modification: numbers that are inconvenient for mental calculation. According to P.Ya. Galperin, such tasks cannot be considered substantial modifications; the “real” modifications should concern changes in the actions required rather than the material itself.

“Coal” task (№ 7) (DS): 12 g of coal produce 396 calories during combustion. It takes 12276 calories to boil a kettle. How much coal will it take to burn?

“Psychological” modification prevails in the “paint” task: students are prompted to use an additive strategy instead of a multiplicative (to add several jars, instead of using proportion). The modifications of this kind are most sensitive to the quality of students’ “conceptual” actions.

“Paint” task (& 2), direct proportion, modified conditions (DM): Some children decided to paint the tribunes on the school stadium green, but they had only yellow (y) and blue (b) paints. Each student took several jars of yellow and blue and poured them together to make some green colour. Here are their paints:

Alice: y y b b b Gregory: y y y b b b b b John: y y b b b b Mike: y b b

Nike: y y y y b b b b b b Tim: y y y b b b b b b Irene: y y y y b b b b b

John began to paint his bench, but the paint ran out before he completed it. Does anyone have a mixture of the same shade to help John complete his bench?

In the “lawn” task students were prompted to use excessive data (the number of seeds needed to grow a regular lawn), so the problem involved both kinds of modification, logical and psychological. The description was lengthy, and the sequence of data presentation itself prompted students toward the wrong solution, since the extra data was introduced first.

“Lawn” task (№ 8) (DM): To make a perfect lawn you have to prepare the soil, water and mow the lawn, and foremost, plant the seeds properly. The instruction says to plant one packet of seeds (120 g) over 4 square meters for a regular lawn, and for a beautiful dense lawn the same number of seeds should be spread over 3 square meters. Ivan Ivanovich decided to make a beautiful dense lawn in front of his house with an area of 24 square. How many grams of seeds does he need to sow?

The “pie”-task has subject-modification: part-whole relations had to be considered in addition to proportion. The task also demands comprehension that the weight of the pieces depends on both “sizes” — length and width.

“Pie” task №10, inverse proportion, modified (IM): A big pie was cut in several pieces. One rectangular piece is 18 cm long and 12 cm wide. The other rectangular piece is 6 cm longer than the first one but weighs the same. “That’s impossible!”- said Serge in surprise. *What reply do you agree with?*

## Results

The students’ written work (160 completed forms) was analyzed: their performance on the sample and modified tasks was compared, and their notes (if any) were examined as indirect evidence of their reasoning.

The α-Cronbach difference for all fifteen tasks — 0.870 — indicates the internal consistency of the parameters of the diagnosis itself.

**Table 1:** 5th and 6th grade students’ performance in sample and modified proportional tasks

	Direct proportion		Inverse proportion	
	Sample tasks	Modified tasks	Sample tasks	Modified tasks
5th grade	82.4	62.1	65.4	46.7
6th grade	86.5	66.2	67.8	45.5

There was no significant difference between the results of the 5th and 6th graders (F-test); thus, for further analyses we considered all 160 tests together.

**Figure 1.**
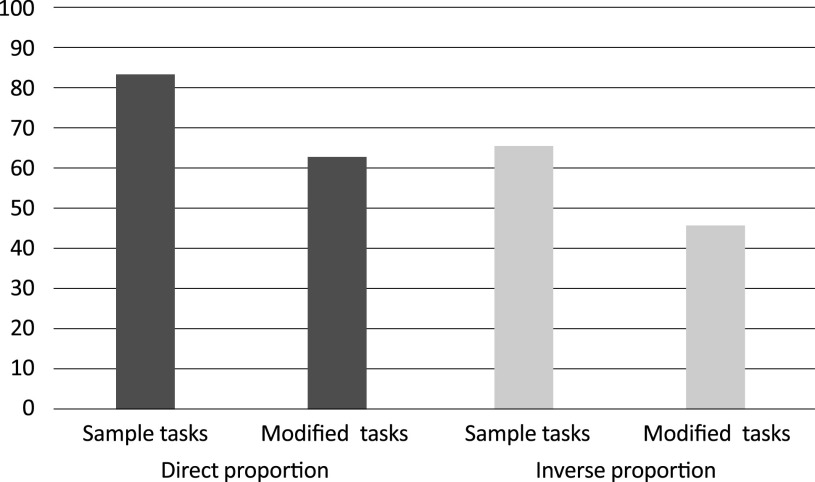
Students’ performance in sample and in modified tasks

*Note. The differences between students’ performance on the sample and modified tasks are significant for both types of proportion (p < 0.01, F-test). The differences between performance in direct-proportion and inverse-proportion tasks are also significant (p < 0.01, F-test).*

*Figure 1* below compares the students’ performance on the sample-tasks and the modified tasks.

The results showed that students’ operations with ratio concepts are sensitive to task variations. Tasks with “unusual” wording, excessive data, and a discrepancy between their visual and conceptual features yielded significantly worse results than regular (sample) tasks (p < 0.01) in both cases: for direct and inverse proportions.

**Figure 2.**
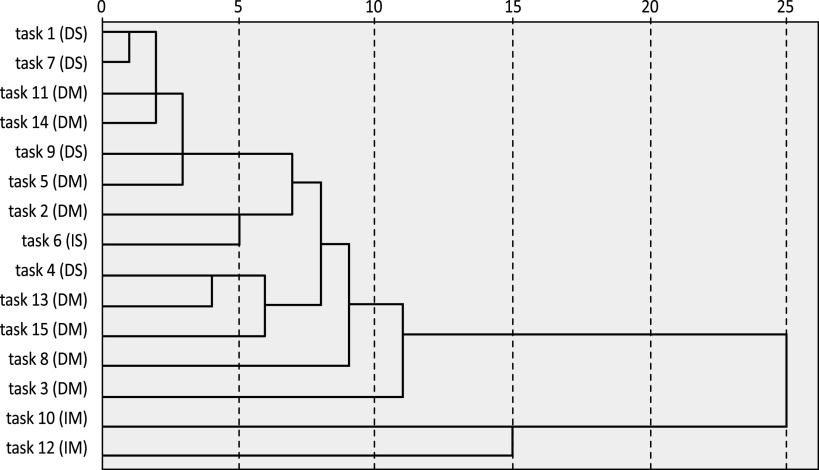
Students’ performance: hierarchical cluster analysis of students’ mistakes

Hierarchical cluster analysis was carried out to evaluate the “similarity” in students’ performance across different tasks. The dendrogram is shown below (*Figure 2*).

The dendrogram allows us to analyze the tasks on which students performed similarly. One cluster can be distinguished among all tasks: the two tasks on inverse proportion (№10, the “pie” task, and the similar №12). Both tasks included variations and yielded significantly worse results than the others. At the same time the “sample” task featuring inverse proportions (task 6) was solved with the same level of competence as those with modified direct proportions. The necessity of considering the changes of two magnitudes with “opposite” arithmetic operations -- if one value is to be “multiplied,” the other is to be “divided” — leads to well-known difficulties and mistakes in choosing the appropriate operations.

The “Coal” task (№7) was solved almost as well as the “shell” task (№1) (they both were the sample-tasks for direct proportions). As was mentioned above, this task was modified in only one aspect: the numbers were huge (3-5 digits) and did not allow mental calculation. As we see, this kind of modification had no significant influence on the students’ performance. This result confirms Galperin’s remark when he addressed educational designers: Changing tasks in the aspect of numbers only is not an essential modification, since it does not fundamentally affect the actions. However, in tasks with “small” numbers, but modified in the way the data is presented (a diagram of the ratio between salt and water in the DM task [№11], or a photo showing the sample portion of buttons in the DM task [№14]), the students’ performance slightly worsened.

The most significant worsening of performance was observed in task №3 (DM) and the “lawn” task (№8) (DM), both of which contained extra data that either prompted wrong calculations or masked the right calculation by excessive wording. The result was that the text itself contained no hints of the sequence of arithmetic operations required.

## Discussion

The comparison of the results shows that the students were more successful on tasks that used direct proportions, and less successful on tasks concerning inverse proportions (p<0.01, F-test). Moreover, students’ performance worsened if the tasks contained modifications which “disguised” the typical calculation: extended wording, excessive data, and use of diagrams to present necessary information.

The most frequent mistakes made by the students were the following:

1. Applying an additive strategy instead of a multiplicative one. Here are some examples:

“Salad” task №5 (DM). According to the recipe, two boiled potatoes and three eggs are needed to make a salad. The cook wants to make the salad exactly by the recipe, but using nine eggs.

“You need to boil eight potatoes, — says the home Elf. — Because potatoes should be one less, than the eggs!”

“No! — argues the greedy Mouse. — Eight potatoes are too much! Six will be enough!”

How many potatoes should he prepare?

Student E. writes: *The home Elf is right! 3–2 = 1; 9–1 = 8*

“Train” task №4 (DS) The train covers 35 km in 25 minutes. What distance will the train cover in 35 minutes?

Student U. writes: *35–25 = 10; 35+10 = 45 kilometers*

Student A. writes: 1) 35–25 = 10 (minutes) — difference

*2) 10⋅35 = 350 (km)*

In the “shells” task (DS) student D. writes: *21 shells!*

In the paint task (see above) the same student D. chooses Nike’s paint (yyyy bbbbbb), which is plus two yellow and plus two blue measures, as compared to John’s paint (yy bbbb).

“Pencil” task ⋅9 (DS). Six copybooks cost as much as 16 pencil sets. John has enough money to buy only 15 copybooks. How many pencil sets he would be able to buy with his money?

Student I. writes: *16-6=10; 15+10 = 25* (pencil sets)

The additive strategy was also used for inverse proportion. In the “pie” task (IM) student K. writes: *If the second piece is 6 cm longer, it means, it is less in width exactly for this 6 cm — then it will weigh the same!*

2. Considering only one magnitude’s changes with no reference to the changes of the other. Here are some examples:

For the coal task (see above) student C. writes: *12276:396=31 (g) *— and considers this to be the answer.

For task 1 (see above) student J. writes: *20:4=5 (shells)*

On the “paint” task, student V. writes: *yy bbbb; yy bbb*, and answers that *John should take Alice’s paint*! Evidently, student V. relies on only one of the parameters for comparison. “Alice” was one of the most popular answers among incorrect replies.

On the “salad” task student T. writes: *8 (crossed out) 6 potatoes are needed. 8 potatoes in one salad are too much!*

The students sometimes made peculiar mistakes (regardless of the task type) which were not substantially connected to any meaning of the problem itself. These solutions looked as if students were merely applying familiar manipulations to the numbers in the task:

Student B. (in the “train” task) writes: *35 .35 = 1225 (km) — it will cover;* Student F. (in the “coal” task) writes: *12276 :12 = 1023*; and the like.

The analysis of the results we obtained shows that the majority of students solved the “sample” tasks, which meant that they were able to operate values in a multiplicative relationship based on their usual school math training. When the modifications (especially psychological ones) were introduced, their orientation proved to be fragile. Here is an illustration from one of the diagnostic lessons: one of the students was solving the “salad” task and writing his answer, which was correct (6 potatoes). The teacher was passing by and asked him to write down the solution or some reasoning. The student looked again at the task and said: *“No, it is reasonable to prepare 8 potatoes indeed! It’s more logical!”* — and “corrected” his answer.

Thus, the modifications, especially the logical and psychological ones, significantly impaired the students’ performance. These modifications did not allow students to “guess” the familiar arithmetic operations according to the formal features of a text-problem (by its very wording and the numbers used). The necessity of reconstructing the substantial relations between the magnitudes to solve the problems became clear, as students failed even simple tasks when they were being distracted by an unusual format (long texts or diagrams).

The aim of this research was to use Galperin’s and Talyzina’s approach to designing diagnostic tasks which are sensitive toward the quality of the students’ orientation underlying their ratio-based problem-solving.

Our analysis of the students’ performance shows that their ability to apply the concepts was very sensitive to the way the problem’s text was presented. Thus, in studying the acquisition of ratio concepts, we have to take into account that its actual level may be disguised by the students using “roundabout” ways to retrieve correct solutions from some random features which depend on the format in which the task is presented. The effects which we observed are typical and indicate the deficiency of generalization and other qualities of the action, as Galperin specified (Galperin, 1992, cited by Engeness, 2020a). The students’ orientation for operating and transforming two magnitudes appears to have been invalid (an orientation they may have gained from previous learning experience): the students failed to consider the change of both values simultaneously, taking only one parameter’s changes into account.

Our long-term goal is to design sound teaching strategies and educational materials, including digital support, to scaffold the means for formation of the consistent ratio concept. Thus, it is necessary to rely on the appropriate diagnostic tools to compare the results of innovative instructions to the results of traditional teaching. The current study was the first step in our attempt to address the problem of teaching multiplicative thinking comprehensively.

The principles of the research strategy which were introduced and grounded by P.Ya. Galperin and N.F. Talyzina, provide researchers with the diagnostic approach to reveal the actual level of new concepts’ development. The “psychological” qualities of action — the consciousness, reasonability, generalization, and critical attitude — are more important than the external efficiency of the executive action performed in sample familiar tasks.

## Conclusion

The analysis of the students’ mistakes was most crucial, since they pointed out the deficits in the orientation basis for solving problems in this domain. In addition to analyzing the mistakes made, one has to question what the correct solutions relied on? Did the students’ solutions imply a conscious, sensible reasoning, or was there some “roundabout” method they used to get the right answer? It is obvious that one cannot rely entirely on students’ success in regular tasks to evaluate the quality of their mastery of mathematical concepts. There can be ways of solving problems without adequate concepts, which ways will fail students whenever the tasks deviate from the typical ones. These issues demand future detailed research.

The findings of this study may be adopted by teachers and education designers to assess the quality of ratio-based concepts acquired by students during the transition period from primary to secondary education, since the mastery of these concepts is essential for students’ success in the Math, Physics, and Chemistry disciplines.

## Limitations

Some of our tasks proved to be more sensitive than others; thus, for future research we plan to focus on the most critical tasks, while the number of “sample” control tasks may be reduced. The diagnostics presented in the study were limited mostly to students’ answers; unfortunately, students often did not explicitly provide their reasoning despite the teacher’s request. Thus, the “questioning” part should be improved to prompt argumentation from participants.

Students’ poor performance on the inverse proportion tasks demands separate research on the psychological conditions for the formation of the corresponding concepts.
